# Rhein Induces Oxidative Stress and Apoptosis in Mouse Blastocysts and Has Immunotoxic Effects during Embryonic Development

**DOI:** 10.3390/ijms18092018

**Published:** 2017-09-20

**Authors:** Chien-Hsun Huang, Wen-Hsiung Chan

**Affiliations:** 1 Department of Obstetrics and Gynecology, Tao-Yuan Hospital, Ministry of Health & Welfare, Taoyuan City 33004, Taiwan; lithsunh@gmail.com; 2 Department of Bioscience Technology and Center for Nanotechnology, Chung Yuan Christian University, Taoyuan City 32023, Taiwan; 3 Department of Medical Research, China Medical University Hospital, China Medical University, Taichung 40402, Taiwan

**Keywords:** rhein, apoptosis, oxidative stress, embryonic development, immunotoxicity

## Abstract

Rhein, a glucoside chemical compound found in a traditional Chinese medicine derived from the roots of rhubarb, induces cell apoptosis and is considered to have high potential as an antitumor drug. Several previous studies showed that rhein can inhibit cell proliferation and trigger mitochondria-related or endoplasmic reticulum (ER) stress-dependent apoptotic processes. However, the side effects of rhein on pre- and post-implantation embryonic development remain unclear. Here, we show that rhein has cytotoxic effects on blastocyst-stage mouse embryos and induces oxidative stress and immunotoxicity in mouse fetuses. Blastocysts incubated with 5–20 μM rhein showed significant cell apoptosis, as well as decreases in their inner cell mass cell numbers and total cell numbers. An in vitro development assay showed that rhein affected the developmental potentials of both pre- and post-implantation embryos. Incubation of blastocysts with 5–20 μM rhein was associated with increased resorption of post-implantation embryos and decreased fetal weight in an embryo transfer assay. Importantly, in an in vivo model, intravenous injection of dams with rhein (1, 3, and 5 mg/kg body weight/day) for four days resulted in apoptosis of blastocyst-stage embryos, early embryonic developmental injury, and decreased fetal weight. Intravenous injection of dams with 5 mg/kg body weight/day rhein significantly increased the total reactive oxygen species (ROS) content of fetuses and the transcription levels of antioxidant proteins in fetal livers. Additional work showed that rhein induced apoptosis through ROS generation, and that prevention of apoptotic processes effectively rescued the rhein-induced injury effects on embryonic development. Finally, the transcription levels of the innate-immunity related genes, *CXCL1*, *IL-1 β* and *IL-8*, were down-regulated in the fetuses of dams that received intravenous injections of rhein. These results collectively show that rhein has the potential to induce embryonic cytotoxicity and induce oxidative stress and immunotoxicity during the development of mouse embryos.

## 1. Introduction

Rhein is a glucoside found among the major chemical compounds of the roots of rhubarb (*Rheum palmatum* L. or *Rheum tanguticum* Maxim) [1]. Previous reports have demonstrated that rhein has various pharmacological effects, including anti-inflammatory [2], anti-allergic [3], antifungal [4], antibacterial [5], antiviral [6] and anticancer [7,8,9] actions. Further studies revealed that rhein could inhibit cell growth or induce cell death in many cancer types, such as human breast cancer [10], cervical cancer [11], gastric cancer [12], and human hepatocellular carcinoma [13]. Recently, rhein was shown to trigger cell apoptosis through mitochondrial-dependent pathways or ER stress-associated cell death processes [14]. These reports showed that rhein acts as a potent inducer of apoptosis. However, although our studies have consistently demonstrated that natural chemical compounds can negatively affect mouse embryonic development by inducting cell apoptosis [15,16,17,18,19,20], the potential cytotoxic effects of rhein on embryonic development have not yet been studied.

Apoptosis plays important roles during normal embryogenesis. It is tightly regulated: apoptotic processes clear redundant or abnormal cells during embryonic development [21,22], but are not seen at earlier (i.e., zygote to blastocyst) developmental stages [23]. Importantly, our group has shown that the induction of cell apoptosis by physical or chemical teratogens in early-stage embryos can negatively impact embryonic development [20,24,25]. However, excessive or unsuitable apoptosis triggered in early embryos can cause injury effects on pre- and post-implantation embryonic development [17,19,20].

A previous study reported that pre-treatment of zebrafish embryos with a pretilachlor for four days affects embryo development [26]. In addition, pretilachlor exposure induces a significant increase in levels of reactive oxygen species (ROS), transcription and antioxidant proteins (catalase, superoxidase dismutase and glutathione peroxidase) in zebrafish. Transcription of CXCL-C1C, IL-1 β and IL-8 related to innate immunity was shown to be downregulated after pretilachlor exposure [26]. These results imply that the pretilachlor can simultaneously induce endocrine disruption, oxidative stress increase and immunotoxicity during zebrafish embryo development. A number of studies have shown that bi-directional interactions connect oxidative stress with apoptosis and immunity [27,28], and apoptosis and oxidative stress affect cytokine responses of lymphocytes as well as apoptosis of immune cells [29,30]. However, no previous investigations to date have examined potential bi-directional interactions of oxidative stress, apoptosis and immune systems in response to rhein. In this study, we evaluated whether in vivo rhein exposure is associated with a long-term decrease in mRNA levels of innate immune-related genes along with a long-term increase in ROS content and transcription levels of antioxidant enzymes, and further determined whether rhein carries a risk for injury of normal embryonic and fetal development.

Here, we investigated how rhein impacts early embryonic development in mouse blastocysts. Our results suggest that rhein causes developmental injury to blastocysts in vitro and in vivo and triggers oxidative stress and immunotoxicity during the pre- and post-implantation development of mouse embryos.

## 2. Results

### 2.1. Effects of Rhein on Mouse Blastocysts

**Figure 1:**
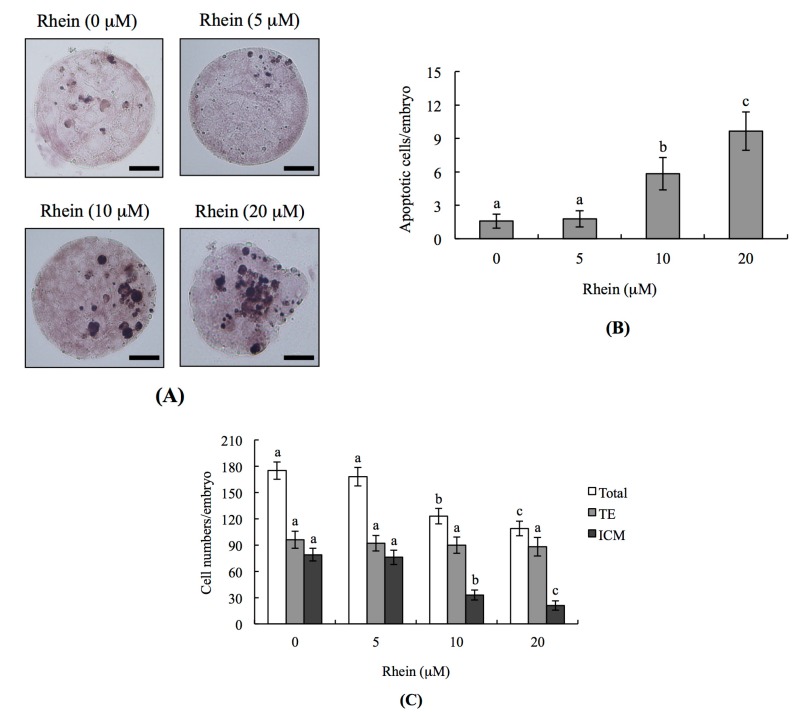
Effects of rhein on mouse blastocysts: (**A**) mouse blastocysts were treated with or without rhein (5, 10 or 20 μM) for 24 h, and terminal deoxynucleotidyl transferase dUTP nick end labeling (TUNEL) staining was used to measure apoptosis. TUNEL-positive cells (black) were visualized by light microscopy; (**B**) apoptotic (TUNEL-positive) cells were calculated and the numbers of apoptotic cells per blastocyst are depicted; and (**C**) differential staining was performed and the numbers of inner cell mass (ICM) and trophectoderm (TE) cells per blastocyst were counted. Data were obtained from at least 180 blastocysts per group. Values are presented as means ± SD of six determinations. Different symbols indicate significant differences at *p* < 0.05. The scale bar is 20 μm.

To examine whether rhein has toxic effects on embryos, we co-incubated mouse blastocysts with 0–20 μM rhein for 24 h and analyzed DNA fragmentation (a characteristic apoptotic index) by terminal deoxynucleotidyl transferase dUTP nick end labeling (TUNEL) assay. DNA fragmentation was significantly increased in mouse blastocysts treated with 10 and 20 μM rhein (Figure 1A,B), indicating that rhein induces cellular apoptosis in mouse blastocysts.

To analyze the effects of rhein on cell proliferation in embryos and determine whether these effects occurred in cells of the inner cell mass (ICM) and/or trophectoderm (TE), we performed differential staining of embryos. Counting of differentially stained cells revealed that there were significantly fewer ICM cells in 10 and 20 μM rhein-treated blastocysts compared with untreated controls, but that there was no difference in TE cells (Figure 1C). Thus, in blastocyst-stage embryos, rhein appears to inhibit proliferation and induce apoptosis in ICM cells without having any negative impact on TE cells.

### 2.2. Impacts of Rhein on Mouse Embryonic Developmental Potential In Vitro

**Figure 2:**
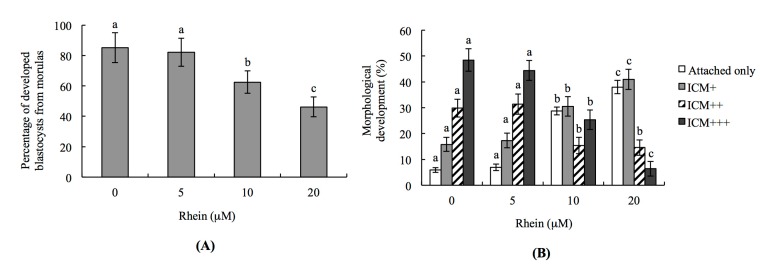
In vitro development of mouse embryos exposed to rhein at the blastocyst stage: (**A**) mouse morulae were treated with or without rhein (5, 10 or 20 μM) for 24 h and cultured for an additional 24 h, and the percentages of blastocysts were calculated; and (**B**) mouse blastocysts were incubated with or without rhein (5, 10 or 20 μM) for 24 h, cultured for seven days, and then classified as attachment-only, ICM+, ICM++, or ICM+++ based on morphological assessment, as described in the Materials and Methods. Values are presented as means ± SD of ten determinations. Data were obtained from 150 blastocysts per group. Different symbols indicate significant differences at *p* < 0.05.

To further investigate the injury effects of rhein on the developmental potential of mouse embryos in vitro, we analyzed the percentage of rhein-treated morulae that developed to blastocysts. Indeed, the percentage of morulae that developed to blastocysts was significantly lower in the 10 and 20 μM rhein-treated groups compared to the untreated control group (Figure 2A).

To test the effects of rhein on post-implantation events in an in vitro development assay, we co-incubated blastocysts with 5–20 μM rhein for 24 h, and then measured their development over an eight-day culture in fibronectin-coated dishes. Importantly, the incubation of blastocysts with rhein was associated with a lower incidence of post-implantation developmental milestones (Figure 2B). Most of the rhein-treated embryos were at the implantation-only stage (attached-only group) or had a lower development score (ICM clustering was scored according to the shapes of the ICM and trophoblastic layer), such as ICM+ (Figure 2B). These results demonstrate that rhein negatively impacts the implantation potential and post-implantation development of mouse embryos in vitro.

### 2.3. Effects of Rhein on Blastocyst Developmental Potential In Vivo

**Figure 3:**
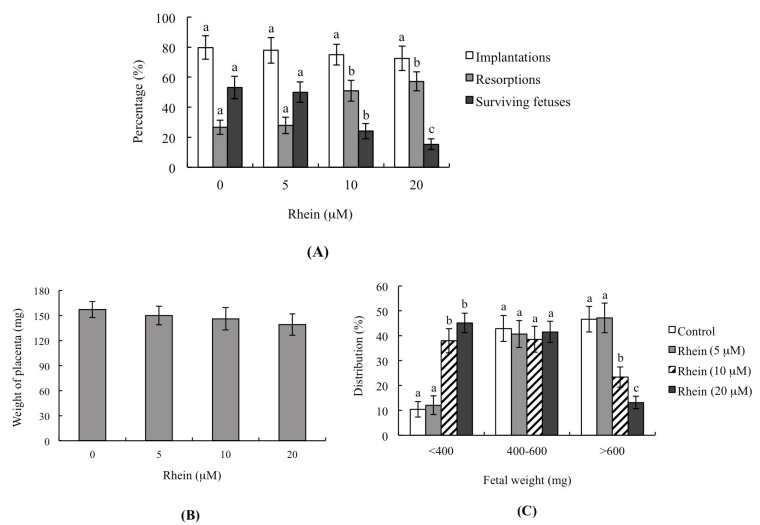
Effects of rhein on the in vivo implantation, resorption, fetal survival and fetal weight of mouse blastocysts. Mouse blastocysts were treated with or without rhein (5, 10 or 20 μM) for 24 h, and embryo transfer was performed as described in the Materials and Methods. (**A**) The percentages of implantation, resorption, and surviving fetuses (i.e., the number of a given event per the number of transferred embryos × 100) were calculated; (**B**) Placental weights of 40 recipient mice were measured; (**C**) Weight distribution of surviving fetuses on Day 13 post-transfer (Day 18 post-coitus). Surviving fetuses were obtained by embryo transfer of 320 total blastocysts across 40 recipients. Different symbols indicate significant differences at *p* < 0.05.

To analyze the impacts of rhein on blastocyst development in vivo, we incubated blastocysts with or without 5–20 μM rhein for 24 h, performed uterine transfer, and then examined the uterine contents at 13 days post-transfer (Day 18 post-coitus). The implantation ratios were significantly lower in the 10 and 20 μM groups compared to the control, but no such difference was seen in the 5 μM group (Figure 3A). The ratios of embryos that implanted but failed to develop and underwent subsequent resorption were also significantly higher in the 10 and 20 μM groups compared to the control group (Figure 3A). Interestingly, the placental weights derived from rhein-pretreated embryos were not significantly different from those of the untreated control group (Figure 3B). However, the average fetal weight, which is considered an important indicator of developmental status [20,31,32,33,34], was significantly lower in the 10 and 20 μM rhein-treated groups compared to the control group (Figure 3C). The embryo transfer assay showed that 46.7% fetal weight of the control group was >600 mg and 42.9% fetal weight was 400–600 mg. The fetal weights of 10 and 20 μM rhein-treated groups were significantly lower relative to the untreated control group. Only 13.2% and 23.4% fetal weights were >600 mg in the 20 μM and 10 μM rhein-treated groups, respectively, clearly indicating that rhein has the potential to cause post-implantation development injury. The percentage of fetal weights <400 mg was 10.4% in the control group and 45.2% in the 20 μM rhein-treated group. The collective results confirm poorer fetal development in the rhein-treated (<400 mg) than the control group. Notably, the average weights of fetuses weighing more than 600 mg (which we previously showed to be an important threshold for successful embryonic and fetal development) were significantly lower in the 10 and 20 μM rhein-treated groups compared to the untreated control group (Figure 3C). These results show that pre-treatment of mouse blastocysts with rhein can reduce their implantation and post-implantation development potential in vivo.

**Figure 4:**
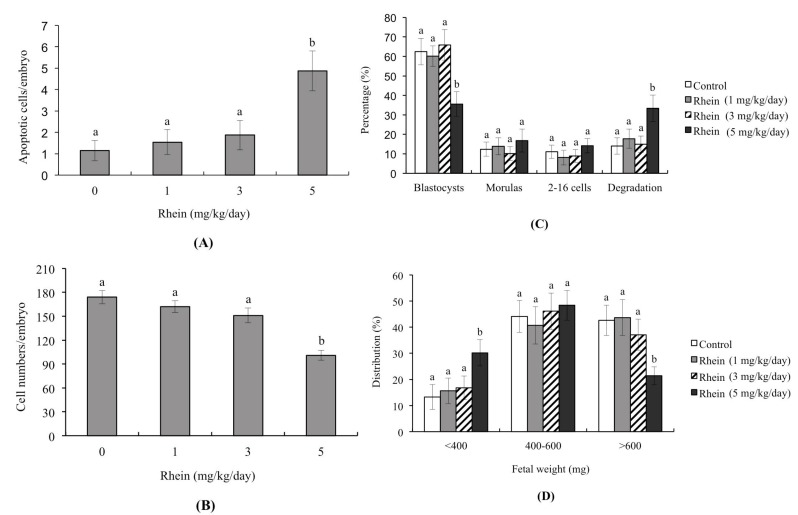
Effects of intravenously injected rhein on apoptosis and blastocyst development in an animal model. Twenty randomly selected female mice were intravenously injected with or without rhein (0, 1, 3, and 5 mg/kg/day). One day (24 h) later, these female mice were mated overnight with a single fertile male of the same strain, and then intravenously injected with rhein (0, 1, 3, and 5 mg/kg/day) daily for four days. Blastocysts were collected by flushing the uterine horn on Day 4 after mating. (**A**) Apoptosis of mouse blastocysts was measured by TUNEL staining followed by light microscopy, and the mean number of TUNEL-positive (apoptotic) cells per blastocyst was calculated; (**B**) Total cell numbers per blastocyst was counted; (**C**) Developmental stages of embryos were examined by flushing the mouse uterine horn with buffer on Day 4. Data are presented as the percentage of total embryos; (**D**) Distribution of surviving fetuses according to weight on Day 18 post-coitus. Data were obtained from 150 blastocysts per group. Different symbols indicate significant differences at *p* < 0.05.

We further examined the possible injury effects of rhein on blastocyst development in an animal model by intravenously injecting female mice with rhein (0, 1, 3, and 5 mg/kg/day). Our results revealed that the blastocysts of mice injected with 5 mg/kg/day rhein showed significantly more apoptosis and significantly less cell proliferation than those of untreated control mice (Figure 4A,B). Moreover, the uterine contents of 5 mg/kg/day rhein-injected animals exhibited decreased embryonic development from the zygote to blastocyst stages, increased embryonic degradation, and decreased fetal weight compared to those of untreated control mice (Figure 4C,D). The percentages of fetuses weighing >600 mg were 21.4% in the 5 mg/kg/day rhein-injected group compared to 42.6% in the untreated control group (Figure 4D). Collectively, our findings suggest that rhein has the potential to reduce successful implantation and decrease post-implantation development in vitro and in vivo.

### 2.4. Effects of Antioxidants and Caspase Inhibitors on the Development of Rhein-Treated Blastocysts

**Figure 5:**
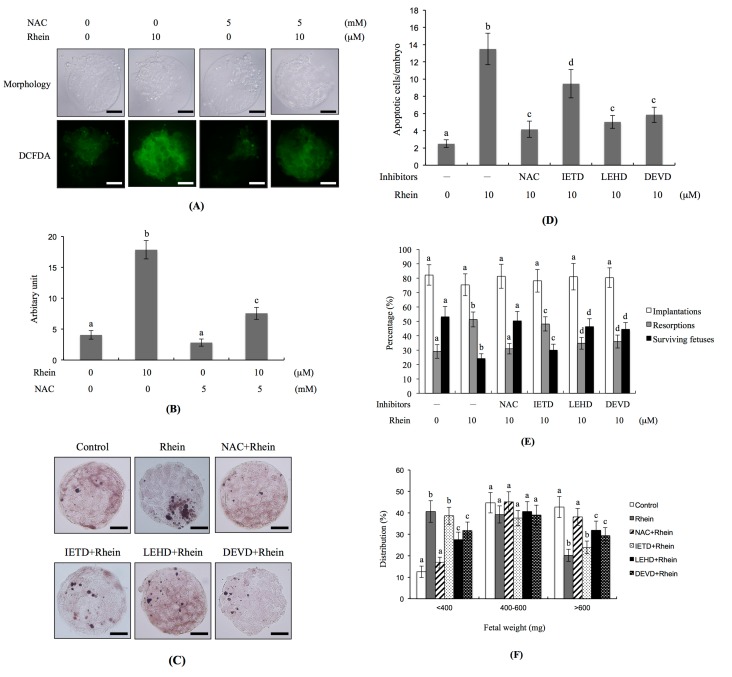
Effects of NAC and caspase inhibitors on the development of rhein-treated blastocysts. Mouse blastocysts were pre-treated with 5 mM *N*-acetyl cysteine (NAC), 300 μM Z-IETD-FMK (IETD), 300 μM Z-LEHD-FMK (LEHD) or 300 μM Z-DEVD-FMK (DEVD) for 1 h or left untreated, and then incubated with or without rhein (10 μM) for a further 24 h. (**A**) Reactive oxygen species (ROS) generation was measured by staining with 20 μM DCF-DA fluorescence dye; (**B**) Intracellular ROS production in each group was quantitatively analyzed using the Image J software; (**C**) Apoptosis was examined by TUNEL staining; (**D**) Mean number of apoptotic (TUNEL-positive) cells per blastocyst was counted; (**E**,**F**) Embryo transfer was performed as described in the Materials and Methods. The percentages of implantation, resorption, and surviving fetuses (i.e., the number of a given event per the number of transferred embryos × 100) were calculated; (**E**) Weight distribution of surviving fetuses on Day 18 post-transfer was measured; (**F**). Control and rhein-pretreated blastocysts were subjected to embryo transfer (a total of 320 blastocysts was transferred to 40 recipients). Different symbols indicate significant differences at *p* < 0.05. The scale bar is 20 μm.

As oxidative stress plays critical roles in the apoptosis triggered by a number of chemical compounds [35,36,37,38,39,40], we investigated whether reactive oxygen species (ROS) could be involved in the rhein-induced apoptosis of blastocysts in vitro. Staining with dichlorodihydrofluorescein diacetate (DCFDA) fluorescence dye (which detects intracellular ROS levels) revealed that the rhein-treated group had significantly higher ROS levels compared to the untreated control group (Figure 5A,B). Conversely, pre-treatment with *N*-acetylcysteine (NAC; a well-known ROS scavenger) effectively prevented this rhein-induced increase in intracellular ROS (Figure 5A,B). Moreover, the TUNEL positivity (indicating apoptosis) of rhein-treated blastocysts was effectively decreased by pretreatment with NAC (Figure 5C,D). Indeed, caspase inhibitors blocked the ability of rhein to increase apoptosis in blastocysts: significant inhibitory effects were seen in blastocysts pretreated with inhibitors of caspase-9 (LEHD) and caspase-3 (DEVD), whereas only partial inhibition was achieved using an inhibitor of caspase-8 (IETD) (Figure 5C,D). In vivo, the developmental failure of rhein-treated blastocysts in our embryo transfer assay was efficiently prevented by pretreatment with NAC or specific inhibitors of caspase-9 or caspase-3 (Figure 5E). The lower fetal weight in the rhein-treated group was significantly rescued by pretreatment of blastocysts with NAC or inhibitors of caspase-9 or caspase-3, but not by the caspase-8 inhibitor (Figure 5F). In mouse blastocyst cells, therefore, rhein appears to trigger a ROS-dependent apoptotic signaling that involves caspase-9 and caspase-3.

### 2.5. Transcription of Genes Related to Innate Immunity and Oxidative Stress in Day-Old Mice Born to Dams Intravenously Injected with Rhein during Pregnancy

**Figure 6:**
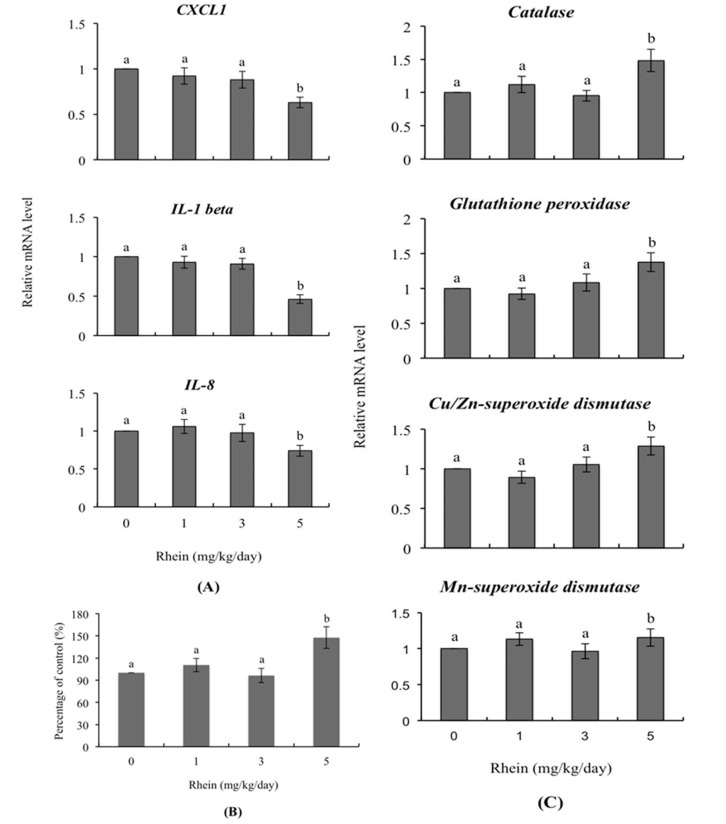
Expression levels of genes related to innate immunity in the livers of day-old mice whose dams were injected with rhein during pregnancy. Twenty randomly selected female mice were intravenously injected with rhein (0, 1, 3, and 5 mg/kg/day) or left untreated. One day (24 h) later, these female mice were mated overnight with a single fertile male of the same strain, and then intravenously injected with rhein (0, 1, 3, and 5 mg/kg/day) for an additional four days. One day after birth, mouse fetuses were collected and cell extracts of fetal liver tissue were prepared. (**A**) The mRNA expression levels of *CXCL1*, *IL-1 β* and *IL-8* were measured using quantitative real-time PCR; (**B**) ROS were detected with 2′,7′-dichlorofluorescin diacetate (DCF-DA) using a fluorescence enzyme-linked immunosorbent assay (ELISA) reader (excitation 530 nm, emission 485 nm); (**C**) The mRNA expression levels of the genes encoding *catalase*, *glutathione peroxidase*, *Cu/Zn superoxide dismutase and Mn superoxide dismutase* were measured. Values are presented as means ± SD of three independent determinations. Data are based on 36 samples from each group. Different symbols indicate significant differences at *p* < 0.05.

The mRNA levels of various genes were detected and were refer to the expression levels of various genes. RT-qPCR was applied to detect the mRNA levels of expression products. To investigate the effects of rhein on immunity-related genes, we detected mRNA levels of *CXCL1*, *IL-1 β* and *IL-8* in day-old mice whose dams had been intravenously injected with rhein (0, 1, 3, and 5 mg/kg/day) during pregnancy. We observed significantly lower levels of these genes among mice born to females that had received 5 mg/kg/day rhein versus untreated control females whereas no such impact was evident in the 1 and 3 mg/kg/day rhein-injected groups (Figure 6A). Importantly, the intracellular ROS content of liver cells from one-day-old mice was significantly higher in the 5 mg/kg/day rhein-injected group compared to those of the untreated control group (Figure 6B). In terms of genes encoding antioxidant proteins, we observed upregulation of the relative mRNA levels of catalase, glutathione peroxidase, Cu/Zn-superoxidase dismutase and Mn-superoxide dismutase in day-old mice of the 5 mg/kg/day treatment group, compared to those of the untreated control group (Figure 6C).

## 3. Discussion

Embryonic development is a precisely regulated and complex process. Numerous environmental factors, chemical agents and physical factors have been shown to exert injury effects on normal developmental processes and lead to malformation or abortion of the embryo. Therefore, it is important to determine the potential teratogenic effects of an apoptotic inducer that has been suggested for therapeutic use. Here, we examined rhein, a glucoside chemical compound found as a component of a traditional Chinese medicine. Rhein has been shown to induce apoptosis among cancer cell lines, and thus may be a good candidate for development as an anti-cancer drug [10,11,12,13]. Rhein reportedly decreases ATP production, reduces the mitochondrial transmembrane potential, up-regulates the release of cytochrome c (Cyto c), and activates caspase-3 to induce apoptosis in Hep-G2 cells; moreover, the mitochondrial permeability transition was found to play a critical role in the rhein-induced cell death of these cells [41]. A recent investigation showed that rhein-induced apoptosis is caspase-dependent and involves ER-stress associated pathways and increased intracellular calcium levels in HL-7702 cells [42]. The same study also found that rhein could induce apoptosis in HL-7702 cells through oxidative stress-involved mitochondria-mediated apoptotic processes. Thus, the previous results collectively demonstrated that rhein is a potent inducer of apoptosis. Here, we further studied whether rhein could cause negative impacts on blastocyst-stage embryos, and thereby affect pre- and post-implantation embryonic development.

Indeed, we found that treatment of mouse blastocysts with rhein for 24 h triggered apoptosis and the loss of ICM cells. Experiments examining the toxicity of rhein (5–20 μM) on pre- and post-implantation embryonic development showed that rhein induces apoptosis, impairs early-stage embryonic development, and has injury effects on post-implantation developmental processes (Figure 1, Figure 2, Figure 3 and Figure 4). Using TUNEL staining to assess DNA fragmentation (an important apoptotic index), we found that rhein dose-dependently triggers apoptosis in mouse blastocysts (Figure 1). Our dual differential staining assay showed that rhein majorly impacted the ICM by triggering a large cell loss, but that no such effect was seen in the TE (Figure 2). Collectively, our study results indicate that rhein (10–20 μM) induces apoptosis to cause a major cell loss in the ICM, and that it negatively affects the pre- and post-implantation embryonic development of mouse blastocysts in vitro and in vivo (Figure 1, Figure 2, Figure 3 and Figure 4).

During normal embryonic development, unwanted cells are eliminated through cell apoptosis; however, apoptosis is not normally seen during the pre-implantation stage, which spans the zygote to blastocyst stages [21,22]. The induction of apoptosis during the pre-implantation stage, such as by physical or chemical injury, could cause damage to or deletion of important cell lineages, thereby impacting pre- or post-implantation embryonic development and potentially leading to embryonic abortion or malformation [23]. During early embryonic development, TE cells contribute to the placenta and are required for development of the mammalian conceptus [43]. Previous studies found that a reduction in the TE cell lineage may reduce embryonic implantation and viability by negatively affecting post-implantation development [34,44,45,46]. Importantly, our study results shown that rhein-induced apoptosis occurs major in ICM but has no negative impacts on TE, reflecting an injury effect on major on pre- and post-implantation embryonic development and fetal development but has minor effects on rate of implantation in vivo by embryo transfer analysis (Figure 3). Thus, rhein-treated blastocysts exhibited decreased embryonic development and increased embryonic death. Recently, coworkers of our team used primary cell cultures derived from mouse ICM and TE to examine the negative impacts of rhein on cells and the underlying regulatory mechanisms. Our preliminary findings suggest that rhein triggers apoptosis in the ICM and TE through the same regulatory mechanism but the treatment dose required to induce negative impacts varies depending on cell type. To further evaluate the in vivo teratogenicity of rhein, we intravenously injected pregnant female mice with rhein and examined the effects of this treatment on the pre- and post-implantation embryonic development of the fetuses. Importantly, our results show that intravenous injection at a dose of 5 mg/kg/day had injury impacts on embryonic development, including increased cell apoptosis, loss of cell numbers, altered pre-implantation embryonic development, and decreased fetal development (Figure 4). The negative impacts of rhein on early embryonic development were evident at doses that reflected physiological concentrations that may be attained via intravenous injection. Thus, although rhein is considered a good candidate for development as an anti-cancer drug that could be delivered in a highly condensed oral tablet or by injection, our results clearly demonstrate that rhein is a potential teratogen that hampers embryonic development in the mouse. Thus, it may be inadvisable for pregnant patients to use rhein as a therapeutic strategy against cancer or another disease. Further investigations are urgently needed to examine the detailed mechanisms responsible for regulating the rhein-induced apoptosis of mouse embryo cells in vivo.

The innate immune system plays critical roles in defense during the early life stages, including fetal stages [47,48]. Previous investigations have shown that a number of environmental and endocrine-disrupting chemical compounds or factors regulate the transcription of cytokines and chemokines and additionally affect the immune system [49,50,51]. Here, we found that the mRNA levels of innate immune-related genes, such as *CXCL-1*, *IL-1 β* and *IL-8*, were significantly reduced in fetuses whose dams were injected with 5 mg/kg/day rhein (Figure 6A). CXCL1, IL-1 β and IL-8 are important chemokines that could attract and activate leukocytes as mediators to induce inflammation responses. Moreover, IL-1 β plays a critical role in activating macrophages and neutrophils at infection sites or tissues [52]. Our findings therefore suggest that injection of dams with 5 mg/kg/day rhein may decrease the immune system activity of fetuses, increasing their susceptibility to infection.

Under normal physiological conditions, intracellular reactive oxygen species (ROS) play critical roles in processes that control cell fate, including cell proliferation and differentiation, by activating transcription factors and a series of signaling cascades [53]. When cells generate excess ROS, the capacity of the redox buffering system to restore the basal level may be exceeded, leading to ROS-triggered damages, such as cell death or aging. ROS can damage cellular biomolecules, such as proteins, DNA and lipids, through oxidation reactions [53]. Various environmental and natural chemical compounds have been shown to disrupt the balance between endogenous and exogenous ROS to induce oxidative stress-triggered damage. The endogenous antioxidative system, which includes various anti-oxidative enzymes (e.g., catalase, glutathione peroxidase and superoxide dismutase), plays important roles in reducing ROS-induced damage and protecting cells against ROS-mediated injury. The protein contents and activities of anti-oxidative enzymes are thus important indicators of the toxic effects of ROS-generating compounds [54,55]. Here, we found that treatment of mouse blastocysts with rhein increased intracellular ROS generation and triggered apoptotic signaling processes that exerted negative impacts on embryonic development. These rhein-induced apoptotic processes were effectively blocked by ROS scavengers, as well as by specific inhibitors of caspase-9 and caspase-3 (Figure 5A–E). Intravenous injection of pregnant mice with rhein significantly increased the ROS content of fetal liver tissues; this was associated with increases in the mRNA levels of the genes encoding catalase, glutathione peroxidase, Cu/Zn superoxide dismutase and Mn superoxide dismutase, which should facilitate detoxification to prevent excess ROS-induced damage (Figure 6B,C). Thus, our study results suggest that rhein induces excess ROS generation and upregulates the transcription of antioxidant enzymes that contribute to ROS elimination.

To our knowledge, no related research to date has established the relationship between dietary rhein consumption and serum concentrations in mammalian models, including rats or mice. To explore this issue, we investigated the effects of several doses of rhein (0, 1, 3, and 5 mg/kg/day) injected intravenously into female mice once daily for four days prior to blastocyst collection. Importantly, intravenous injection with 5 mg/kg body weight/day rhein for four days induced apoptosis of blastocyst-stage embryos, early embryonic developmental injury and decreased fetal weight. The absorption rates of rhein in the intestine and plasma concentrations are yet to be established. Several drug delivery techniques and biomaterials have recently been developed for packing natural chemical compounds with low absorption properties into easily absorbed high-dose formulae for application in constituents of health foods or anticancer drugs [56]. The rhein plasma concentration is believed to easily reach the pharmaceutical dose level via these packing or delivery systems, and it is therefore important to ascertain whether the compound exerts injury effects and/or acts as a teratogen in vivo.

## 4. Materials and Methods

### 4.1. Chemicals and Reagents

Rhein, pregnant mare’s serum gonadotropin (PMSG), bovine serum albumin (BSA), 2′,7′-dichlorofluorescin diacetate (DCF-DA) and sodium pyruvate were purchased from Sigma (St. Louis, MO, USA). Human chorionic gonadotropin (hCG) was obtained from Serono (NV Organon Oss, the Netherlands). The TUNEL in situ cell death detection kit was acquired from Roche Molecular Biochemicals (Mannheim, Germany) and CMRL-1066 from Gibco Life Technologies (Grand Island, NY, USA). TRIzol reagent and the reverse transcriptase kit were purchased from Takara Biomedicals (Kusatsu, Japan).

### 4.2. Mouse Morula and Blastocyst Collection

Institute of Cancer Research (ICR) mice were purchased from Taiwan National Laboratory Animal Center (Taiwan, Republic of China). All experiments were approved by the Animal Research Ethics Board of Chung Yuan Christian University (Taiwan). Humane care was provided for animals following the guidelines for the Guide to The Care and Use of Experimental Animals (Canadian Council on Animal Care, Ottawa, 1993; ISBN: 0-919087-18-3). All mice were maintained on breeder chow (Harlan Teklad) with food and water available ad libitum. Mice were housed in standard 28 cm × 16 cm × 11 cm (height) polypropylene cages with wire-grid tops under a 12 h night/12 h day regimen. Nulliparous females 6–8 weeks old were superovulated via injection of 5 IU PMSG. After 48 h, mice were further injected with 5 IU hCG, followed by mating overnight with a single fertile male of the same strain. The second morning after mating, a vaginal plug was observed, defined as Day 0 of gestation. Plug-positive and -negative females were separated and mice in the positive group used for further experimentation. Morulas were obtained by flushing uterine tubes on the afternoon of gestation Day 3 and blastocysts collected by flushing the uterine horn on Day 4. The flushing solution for collection of morulas or blastocysts consisted of CMRL-1066 medium containing 1 mM glutamine and 1 mM sodium pyruvate. Expanded embryos (morulas or blastocysts) obtained from different females were pooled and randomly selected for further experiments.

### 4.3. Analysis of Blastocysts Developed from Morulas

Morulas were co-incubated with rhein (5, 10 or 20 μM) for 24 h or left untreated and cultured for an additional 24 h at 37 °C for embryo development. Numbers of blastocyst-stage embryos were counted and the percentages of morulas developing into blastocysts determined using phase-contrast microscopy (Olympus BX51, Tokyo, Japan).

### 4.4. TUNEL Assay of Rhein-Treated Embryos

Rhein stock solution is dissolved in dimethyl sulfoxide (DMSO) and stored at −30 °C. For the experiments, rhein was added to embryo culture medium at the indicated concentrations with a final DMSO concentration of up to 0.5% (*v*/*v*). Blastocysts were incubated with 0.5% DMSO as the control (or vehicle group) or medium containing 5–20 µM rhein for 24 h. Blastocysts were co-incubated in medium containing rhein (0–20 μM) for 24 h. For detection of DNA fragmentation, an important parameter of apoptosis, embryos were washed in rhein-free medium, fixed in 4% paraformaldehyde (PFA) at room temperature for 2 h, permeabilized and subjected to TUNEL labeling using an in situ cell death detection kit (Roche Molecular Biochemicals) according to the manufacturer’s protocol. In brief, each group of blastocysts was incubated with 20 μL TUNEL reaction mixture (2 µL enzyme solution and 18 µL labeling solution containing fluorescein-conjugated nucleotides) for 30 min at 37 °C. Blastocysts were washed three times with phosphate-buffered saline (PBS) containing 0.3% (*w*/*v*) BSA to remove the TUNEL reaction mixture. Each group of embryos was incubated with converted peroxidase (POD) solution (20 µL) for 30 min at 37 °C. Embryos were extensively re-washed with PBS and finally incubated with 20 µL DAB (3,3’-diaminobenzidine) substrate solution for 3 min at room temperature. Images were obtained via fluorescence microscopy under bright light.

### 4.5. Cell Proliferation in Rhein-Treated Embryos

Blastocysts were incubated with or without culture medium containing 5, 10 or 20 μM rhein for 24 h. Embryos were further washed with rhein-free medium and subjected to dual differential staining to facilitate counting of cell numbers in the inner cell mass (ICM) and trophectoderm (TE) according to a previous study [44]. For removal of the zona pellucida of embryos, blastocysts were incubated in 0.4% pronase in M_2_–BSA medium (M_2_ medium containing 0.1% bovine serum albumin). Denuded blastocysts were exposed to 1 mM trinitrobenzenesulphonic acid (TNBS) in BSA-free M_2_ medium containing 0.1% polyvinylpyrrolidone (PVP) at 4 °C for 30 min and washed with M_2_ medium, in keeping with a previous report [57]. Next, blastocysts were incubated with 30 μg/mL anti-dinitrophenol-BSA complex antibody in M_2_-BSA at 37 °C for 30 min and co-incubated with M_2_ medium supplemented with 10% whole guinea pig serum containing complement, along with 20 μg/mL bisbenzimide and 10 μg/mL propidium iodide (PI), at 37 °C for 30 min. Immunolysed blastocysts were gently transferred to slides and protected from light before observation under a fluorescence microscope. Under UV light excitation, ICM cells (which take up bisbenzimidine but exclude PI) appeared blue whereas TE cells (which take up both fluorochromes) appeared orange-red. As multinucleated cells are not common in preimplantation mouse embryos, nuclear numbers were considered to represent an accurate measure of cell number [58].

### 4.6. Embryonic Development via Morphological Analysis

Blastocysts were cultured in vitro according to a modification method reported in a previous study [59]. In brief, four embryos were cultured per well in fibronectin-coated 4-well multidishes at 37 °C. The basic medium for embryo culture consisted of CMRL-1066 supplemented with 1 mM glutamine and 1 mM sodium pyruvate plus 50 IU/mL penicillin and 50 mg/mL streptomycin (hereafter designated “culture medium”). In the treatment groups, embryos were incubated with 5–20 μM rhein for 24 h in serum-free medium. Thereafter, rhein-treated embryos were cultured for three days in medium supplemented with 20% fetal calf serum and four days in medium supplemented with 20% heated-inactivated human placental cord serum for a total culture time of eight days from the onset of treatment (including the rhein treatment duration time of 24 h). The embryo development status was inspected daily under a phase-contrast dissecting microscope, and developmental stages classified according to established criteria [60,61]. Under these culture conditions, each hatched blastocyst attached to fibronectin and grew to form a cluster of ICM cells over the trophoblastic layer via a process known as TE outgrowth. Morphological scores for embryo outgrowth were estimated over a total incubation period of eight days. Growing embryos were classified as either “attached” or “outgrowth”, the latter being defined by the presence of a cluster of ICM cells over the trophoblastic layer. As described previously, ICM clusters were scored based on shape, ranging from compact and rounded (+++) to a few scattered cells (+) over the trophoblastic layer [62,63].

### 4.7. Blastocyst Development Following Embryo Transfer

Embryo transfer was performed with a nonsurgical embryo transfer (NSET) device for transfer of blastocysts according to a previously reported method [64]. For further investigation of the ability of expanded blastocysts to implant and develop in vivo, generated embryos were treated with or without rhein and transferred to 40 pseudopregnant recipient mice. ICR females (white skin color) were mated with vasectomized males (C57BL/6J; black skin color; from National Laboratory Animal Center, Taiwan) to produce pseudopregnant dams. One vasectomized fertile male mouse was used to mate one ICR female and a total of 40 pseudopregnant dams produced as recipients for embryo transfer. Skin color was evaluated at Day 18 post-coitus to ensure that all fetuses in pseudopregnant mice were obtained from embryo transfer (white color) and not fertilization by C57BL/6J (black color). To examine the impact of rhein on postimplantation growth in vivo, blastocysts were treated with 0, 5, 10 and 20 μM rhein for 24 h, and 8 embryos transferred in parallel to paired uterine horns of pseudopregnant mice on Day 4. Surrogate mice were sacrificed on Day 18 post-coitus (Day 13 post-transfer), and the rate of implantation calculated as the number of implantation sites per number of embryos transferred. The incidence rates of resorption and survival were calculated as the number of resorbed or surviving fetuses, respectively, per number of implantations. The weights of surviving fetuses and placenta were measured immediately after sacrifice.

### 4.8. Intravenous Injection of Female Mice with Rhein and Collection of Blastocysts

To examine the impact of rhein on mouse embryonic development in vivo, 20 randomly selected female (42 days old) mice were injected with rhein (0, 1, 3, and 5 mg/kg/day) via intravenous tail injection. After 24 h, injected female mice were mated overnight with a single fertile male of the same strain. Mice were intravenously injected with rhein (0, 1, 3, and 5 mg/kg/day) continuously for four days after mating and blastocysts collected by flushing the uterine horn on Day 4. Cell apoptosis and proliferation of blastocysts and embryonic development were examined.

### 4.9. Pre-Treatment of Blastocysts with NAC or Caspase Inhibitors

Mouse blastocysts were collected by flushing the uterine horn on Day 4 and either left untreated or pre-treated with 5 mM *N*-acetyl cysteine (NAC), 300 μM Z-IETD-FMK (IETD), 300 μM Z-LEHD-FMK (LEHD) or 300 μM Z-DEVD-FMK (DEVD) for 1 h followed by treatment with rhein (5, 10 or 20 μM) for 24 h. The DCF-DA fluorescence dye was used to detect and measure ROS generation. Quantitative analysis of intracellular ROS production in each group was conducted with Image J software (National Institutes of Health, Rockville, MD, USA).

### 4.10. Gene Expression and ROS Content in Fetal Liver

Twenty randomly selected female mice were intravenously injected with rhein (0, 1, 3, and 5 mg/kg/day) or left untreated. After 24 h, female mice were mated overnight with a single fertile male of the same strain and intravenously injected with rhein (0, 1, 3, and 5 mg/kg/day) for four days. One day after birth, ten mouse fetuses were collected. Cell extracts of fetal liver tissue were prepared via homogenization with ice-cold phosphate buffered saline (PBS) and lysed in 1 mL homogenization buffer (20 mM Tris-HCl, pH 7.4, 1 mM ethylene-diaminetetraacetic acid (EDTA), 1 mM ethylene-glycol-bis(b-aminoethylether) *N*,*N*,*N′*,*N′*-tetraacetic acid (EGTA), 1% Triton X-100, 1 mM benzamidine, 1 mM phenylmethylsulfonyl fluoride, 50 mM sodium fluoride, 20 mM sodium pyrophosphate and 1 mM sodium orthovanadate). Tissue lysates were collected and centrifuged at 15,000× *g* for 20 min at 4 °C. The resulting supernatant fractions were used as cell extracts. mRNA expression levels of CXCL1, IL-1 β, IL-8, catalase, glutathione peroxidase, Cu/Zn superoxide dismutase and Mn superoxide dismutase were measured using quantitative real-time PCR. ROS arbitrary units were measured using dichlorodihydrofluorescein diacetate (DCFDA) dye. Cell extracts (20 μg) were incubated in 50 μL PBS containing 20 mM DCFDA for 1 h at 37 °C, and relative ROS units determined using a fluorescence ELISA reader (excitation at 530 nm, emission at 485 nm). An aliquot of the cell suspension was lysed for determination of protein concentrations. Results are expressed as arbitrary absorbance units/mg protein.

### 4.11. Analysis of Gene Expression

Cell extracts of ten one-day-old fetal liver tissues were prepared via homogenization. Total RNA was extracted and genomic DNA removed with TRIzol reagent (Life Technologies) and purified with an RNeasy Mini kit (Qiagen, Hilden, Germany), according to the manufacturers’ protocols. Conversion of mRNA into cDNA was performed using the Transcriptor First Strand cDNA Synthesis Kit (Roche, Basel, Switzerland) according to the manufacturers’ protocols. Real-time PCR was performed with an ABI 7000 Prism Sequence Detection System (Applied Biosystems, Foster City, CA, USA) and detection probes using GoTaq^®^ Probe qPCR Master Mix (Promega, Madison, WI, USA). Melt-curve analysis was performed to ensure qPCR data were obtained from single product amplification. β-Actin mRNA levels were quantified as the endogenous control and used for normalization. The following primers were employed for real-time quantitative RT-PCR: CXCL1 (Forward, 5′-TGAGCTGCGCTGTCAGTGCCT-3′ and Reverse, 5′-AGAAGCCAGCGTTCACCAGA-3′), IL-1 β (Forward, 5′-AAGGAGAACCAAGCAACGACAAAA-3′ and Reverse, 5′-TGGGGAACTCTGCAGACTCAAACT-3′), IL-8 (Forward, 5-CACCTCAAGAACATCCAGAGCT-3′ and Reverse, 5′-CAAGCAGAACTGAACTACCATCG-3′), Catalase (Forward, 5′-GCAGATACCTGTGAACTGTC-3′ and Reverse, 5′-GTAGAATGTCCGCACCTGAG-3′), glutathione peroxidase (GPX) (Forward, 5′-CCTCAAGTACGTCCGACCTG-3′ and Reverse, 5′-CAATGTCGTTGCGGCACACC-3′), Cu/Zn-superoxide dismutase (Cu/Zn-SOD) (Forward, 5′-AAGGCCGTGTGCGTGCTGAA-3′ and Reverse, 5′-CAGGTCTCCAACATGCCTCT-3′), Mn-superoxide dismutase (Mn-SOD) (Forward, 5′-GCACATTAACGCGCAGATCA-3′ and Reverse, 5′-AGCCTCCAGCAACTCTCCTT-3′), and β-actin (Forward, 5′-CGTACCACAGGCATTGTGATG-3′, and Reverse, 5′-CTTCTAGGACTGGCTCGCAC-3′).

### 4.12. Statistical Analysis

Data were analyzed using one-way ANOVA, followed by Dunnett’s test for multiple comparisons, and presented as means ± SD. All data were normally distributed and met the assumptions made by the parametric ANOVA test. Different symbols represent significant differences at *p* < 0.05.

## 5. Conclusions

In sum, we herein report for the first time that rhein induces apoptosis in the ICM of mouse blastocysts, decreasing embryonic development and viability both in vitro and in vivo. Rhein triggers blastocyst development injury through ROS-mediated caspase-9- and caspase-3-dependent apoptotic processes. Moreover, in vivo rhein exposure is associated with long-term decreases in the mRNA levels of innate immune-related genes, but long-term increases in the ROS content and transcription levels of antioxidant enzymes. Together, these results demonstrate that rhein carries a risk for injuring normal embryonic and fetal development.

## References

[1] Lee J.H., Kim J.M., Kim C. (2003). Pharmacokinetic analysis of rhein in *Rheum undulatum* L.. J. Ethnopharmacol..

[2] Lee B.H., Huang Y.Y., Duh P.D., Wu S.C. (2012). Hepatoprotection of emodin and Polygonum multiflorum against CCl(4)-induced liver injury. Pharm. Biol..

[3] Singh B., Nadkarni J.R., Vishwakarma R.A., Bharate S.B., Nivsarkar M., Anandjiwala S. (2012). The hydroalcoholic extract of *Cassia alata* (Linn.) leaves and its major compound rhein exhibits antiallergic activity via mast cell stabilization and lipoxygenase inhibition. J. Ethnopharmacol..

[4] Agarwal S.K., Singh S.S., Verma S., Kumar S. (2000). Antifungal activity of anthraquinone derivatives from *Rheum emodi*. J. Ethnopharmacol..

[5] Cyong J., Matsumoto T., Arakawa K., Kiyohara H., Yamada H., Otsuka Y. (1987). Anti-bacteroides fragilis substance from rhubarb. J. Ethnopharmacol..

[6] Barnard D.L., Huffman J.H., Morris J.L., Wood S.G., Hughes B.G., Sidwell R.W. (1992). Evaluation of the antiviral activity of anthraquinones, anthrones and anthraquinone derivatives against human cytomegalovirus. Antiviral Res..

[7] Delpino A., Paggi M.G., Gentile P.F., Castiglione S., Bruno T., Benass M., Floridi A. (1992). Protein synthetic activity and adenylate energy charge in Rhein-treated cultured human glioma cells. Cancer Biochem. Biophys..

[8] Castiglione S., Fanciulli M., Bruno T., Evangelista M., del Carlo C., Paggi M.G., Chersi A., Floridi A. (1993). Rhein inhibits glucose uptake in Ehrlich ascites tumor cells by alteration of membrane-associated functions. Anticancer Drugs.

[9] Lin S., Fujii M., Hou D.X. (2003). Rhein induces apoptosis in HL-60 cells via reactive oxygen species-independent mitochondrial death pathway. Arch. Biochem. Biophys..

[10] Chang C.Y., Chan H.L., Lin H.Y., Way T.D., Kao M.C., Song M.Z., Lin Y.J., Lin C.W. (2012). Rhein induces apoptosis in human breast cancer cells. Evid. Based Complement. Altern. Med..

[11] Ip S.W., Weng Y.S., Lin S.Y., Mei D., Tang N.Y., Su C.C., Chung J.G. (2007). The role of Ca^+2^ on rhein-induced apoptosis in human cervical cancer Ca Ski cells. Anticancer Res..

[12] Li Y., Xu Y., Lei B., Wang W., Ge X., Li J. (2012). Rhein induces apoptosis of human gastric cancer SGC-7901 cells via an intrinsic mitochondrial pathway. Braz. J. Med. Biol. Res..

[13] Shi P., Huang Z., Chen G. (2008). Rhein induces apoptosis and cell cycle arrest in human hepatocellular carcinoma BEL-7402 cells. Am. J. Chin. Med..

[14] Bounda G.A., Zhou W., Wang D.D., Yu F. (2015). Rhein elicits in vitro cytotoxicity in primary human liver hl-7702 cells by inducing apoptosis through mitochondria-mediated pathway. Evid. Based Complement. Altern. Med..

[15] Chan W.H. (2015). Hazardous effects of sanguinarine on maturation of mouse oocytes, fertilization, and fetal development through apoptotic processes. Environ. Toxicol..

[16] Chan W.H. (2014). Cytotoxic effects of dillapiole on embryonic development of mouse blastocysts in vitro and in vivo. Int. J. Mol. Sci..

[17] Chan W.H. (2011). Embryonic toxicity of sanguinarine through apoptotic processes in mouse blastocysts. Toxicol. Lett..

[18] Chang M.H., Huang F.J., Chan W.H. (2012). Emodin induces embryonic toxicity in mouse blastocysts through apoptosis. Toxicology.

[19] Chan W.H. (2007). Citrinin induces apoptosis via a mitochondria-dependent pathway and inhibition of survival signals in embryonic stem cells, and causes developmental injury in blastocysts. Biochem. J..

[20] Chan W.H. (2006). Ginkgolide B induces apoptosis and developmental injury in mouse embryonic stem cells and blastocysts. Hum. Reprod..

[21] Hardy K. (1997). Cell death in the mammalian blastocyst. Mol. Hum. Reprod..

[22] Hardy K., Stark J., Winston R.M. (2003). Maintenance of the inner cell mass in human blastocysts from fragmented embryos. Biol. Reprod..

[23] Byrne A.T., Southgate J., Brison D.R., Leese H.J. (1999). Analysis of apoptosis in the preimplantation bovine embryo using TUNEL. J. Reprod. Fertil..

[24] Chan W.H. (2008). Effects of citrinin on maturation of mouse oocytes, fertilization, and fetal development in vitro and in vivo. Toxicol. Lett..

[25] Chan W.H. (2010). Cytotoxic effects of 2-bromopropane on embryonic development in mouse blastocysts. Int. J. Mol. Sci..

[26] Jiang J., Chen Y., Yu R., Zhao X., Wang Q., Cai L. (2016). Pretilachlor has the potential to induce endocrine disruption, oxidative stress, apoptosis and immunotoxicity during zebrafish embryo development. Environ. Toxicol. Pharmacol..

[27] Jiang J., Wu S., Liu X., Wang Y., An X., Cai L., Zhao X. (2015). Effect of acetochlor on transcription of genes associated with oxidative stress, apoptosis, immunotoxicity and endocrine disruption in the early life stage of zebrafish. Environ. Toxicol. Pharmacol..

[28] Tu W., Niu L., Liu W., Xu C. (2013). Embryonic exposure to butachlor in zebrafish (*Danio rerio*): Endocrine disruption, developmental toxicity and immunotoxicity. Ecotoxicol. Environ. Saf..

[29] Livingstone D.R. (2001). Contaminant-stimulated reactive oxygen species production and oxidative damage in aquatic organisms. Mar. Pollut. Bull..

[30] Murugesan P., Kanagaraj P., Yuvaraj S., Balasubramanian K., Aruldhas M.M., Arunakaran J. (2005). The inhibitory effects of polychlorinated biphenyl Aroclor 1254 on Leydig cell LH receptors, steroidogenic enzymes and antioxidant enzymes in adult rats. Reprod. Toxicol..

[31] Huang F.J., Hsuuw Y.D., Lan K.C., Kang H.Y., Chang S.Y., Hsu Y.C., Huang K.E. (2006). Adverse effects of retinoic acid on embryo development and the selective expression of retinoic acid receptors in mouse blastocysts. Hum. Reprod..

[32] Chan W.H. (2009). Impact of genistein on maturation of mouse oocytes, fertilization, and fetal development. Reprod. Toxicol..

[33] Chan W.H., Shiao N.H. (2008). Cytotoxic effect of CdSe quantum dots on mouse embryonic development. Acta Pharmacol. Sin..

[34] Chan W.H., Shiao N.H. (2007). Effect of citrinin on mouse embryonic development in vitro and in vivo. Reprod. Toxicol..

[35] Chan W.H., Chang Y.J. (2006). Dosage effects of resveratrol on ethanol-induced cell death in the human K562 cell line. Toxicol. Lett..

[36] Chan W.H. (2011). Resveratrol protects against 2-bromopropane-induced apoptosis and disruption of embryonic development in blastocysts. Int. J. Mol. Sci..

[37] Chan W.H., Wu H.J. (2006). Protective effects of curcumin on methylglyoxal-induced oxidative DNA damage and cell injury in human mononuclear cells. Acta Pharmacol. Sin..

[38] Chan W.H., Wu H.J., Hsuuw Y.D. (2005). Curcumin inhibits ROS formation and apoptosis in methylglyoxal-treated human hepatoma G2 cells. Ann. N. Y. Acad. Sci..

[39] Senathilake K.S., Karunanayake E.H., Samarakoon S.R., Tennekoon K.H., de Silva E.D., Adhikari A. (2017). Oleanolic acid from antifilarial triterpene saponins of dipterocarpus zeylanicus induces oxidative stress and apoptosis in filarial parasite Setaria digitata in vitro. Exp. Parasitol..

[40] He G., Feng C., Vinothkumar R., Chen W., Dai X., Chen X., Ye Q., Qiu C., Zhou H., Wang Y. (2016). Curcumin analog EF24 induces apoptosis via ROS-dependent mitochondrial dysfunction in human colorectal cancer cells. Cancer Chemother. Pharmacol..

[41] Du Q., Bian X.L., Xu X.L., Zhu B., Yu B., Zhai Q. (2013). Role of mitochondrial permeability transition in human hepatocellular carcinoma Hep-G2 cell death induced by rhein. Fitoterapia.

[42] KoraMagazi A., Wang D., Yousef B., Guerram M., Yu F. (2016). Rhein triggers apoptosis via induction of endoplasmic reticulum stress, caspase-4 and intracellular calcium in primary human hepatic HL-7702 cells. Biochem. Biophys. Res. Commun..

[43] Cross J.C., Werb Z., Fisher S.J. (1994). Implantation and the placenta: Key pieces of the development puzzle. Science.

[44] Pampfer S., de Hertogh R., Vanderheyden I., Michiels B., Vercheval M. (1990). Decreased inner cell mass proportion in blastocysts from diabetic rats. Diabetes.

[45] Kelly S.M., Robaire B., Hales B.F. (1992). Paternal cyclophosphamide treatment causes postimplantation loss via inner cell mass-specific cell death. Teratology.

[46] Chan W.H. (2005). Ginkgolides induce apoptosis and decrease cell numbers in mouse blastocysts. Biochem. Biophys. Res. Commun..

[47] Lam S.H., Chua H.L., Gong Z., Lam T.J., Sin Y.M. (2004). Development and maturation of the immune system in zebrafish, danio rerio: A gene expression profiling, in situ hybridization and immunological study. Dev. Comp. Immunol..

[48] Trede N.S., Langenau D.M., Traver D., Look A.T., Zon L.I. (2004). The use of zebrafish to understand immunity. Immunity.

[49] Eder K.J., Clifford M.A., Hedrick R.P., Kohler H.R., Werner I. (2008). Expression of immune-regulatory genes in juvenile Chinook salmon following exposure to pesticides and infectious hematopoietic necrosis virus (IHNV). Fish Shellfish Immunol..

[50] Jiang J., Wu S., Wu C., An X., Cai L., Zhao X. (2014). Embryonic exposure to carbendazim induces the transcription of genes related to apoptosis, immunotoxicity and endocrine disruption in zebrafish (*Danio rerio*). Fish Shellfish Immunol..

[51] Jin Y., Zheng S., Fu Z. (2011). Embryonic exposure to cypermethrin induces apoptosis and immunotoxicity in zebrafish (*Danio rerio*). Fish Shellfish Immunol..

[52] Baggiolini M., Moser B., Clark-Lewis I. (1994). Interleukin-8 and related chemotactic cytokines. Chest.

[53] Orrenius S. (2007). Reactive oxygen species in mitochondria-mediated cell death. Drug Metab. Rev..

[54] Risso-de F.C., Orsini N., de Sousa G., Rahmani R. (2004). Cadmium-induced apoptosis through the mitochondrial pathway in rainbow trout hepatocytes: Involvement of oxidative stress. Aquat. Toxicol..

[55] Valavanidis A., Vlahogianni T., Dassenakis M., Scoullos M. (2006). Molecular biomarkers of oxidative stress in aquatic organisms in relation to toxic environmental pollutants. Ecotoxicol. Environ. Saf..

[56] Tsai Y.M., Chang-Liao W.L., Chien C.F., Lin L.C., Tsai T.H. (2012). Effects of polymer molecular weight on relative oral bioavailability of curcumin. Int. J. Nanomedicine.

[57] Hardy K., Handyside A.H., Winston R.M. (1989). The human blastocyst: Cell number, death and allocation during late preimplantation development in vitro. Development.

[58] Gardner R.L., Davies T.J. (1993). Lack of coupling between onset of giant transformation and genome endoreduplication in the mural trophectoderm of the mouse blastocyst. J. Exp. Zool..

[59] Huang F.J., Wu T.C., Tsai M.Y. (2001). Effect of retinoic acid on implantation and post-implantation development of mouse embryos in vitro. Hum. Reprod..

[60] Witschi E. (1972). Characterization of developmental stages. Part II. Rat. Biology Data Book.

[61] Armant D.R., Kaplan H.A., Lennarz W.J. (1986). Fibronectin and laminin promote in vitro attachment and outgrowth of mouse blastocysts. Dev. Biol..

[62] Pampfer S., Wuu Y.D., Vanderheyden I., de Hertogh R. (1994). In vitro study of the carry-over effect associated with early diabetic embryopathy in the rat. Diabetologia.

[63] Huang L.H., Shiao N.H., Hsuuw Y.D., Chan W.H. (2007). Protective effects of resveratrol on ethanol-induced apoptosis in embryonic stem cells and disruption of embryonic development in mouse blastocysts. Toxicology.

[64] Steele K.H., Hester J.M., Stone B.J., Carrico K.M., Spear B.T., Fath -G.A. (2013). Nonsurgical embryo transfer device compared with surgery for embryo transfer in mice. J. Am. Assoc. Lab. Anim. Sci..

